# De novo creation of solid-placenta, few-seeded melon via targeted mutation of *CmSLM/CmAGL11*

**DOI:** 10.1186/s43897-026-00263-6

**Published:** 2026-11-01

**Authors:** Jian Ma, Guoliang Yuan, Huijun Zhang, Weiqi Han, Mengqing Lu, Haonan Cui, Congcong Li

**Affiliations:** 1 Key Laboratory of Vegetable Biobreeding, Key Laboratory of Biology and Genetic Improvement of Horticultural Crops (North China), Ministry of Agriculture and Rural Affairs, Beijing Vegetable Research Center, Beijing Academy of Agriculture and Forestry Sciences (BAAFS), Beijing, 100097 China; 2 School of Life Science, Huaibei Normal University, Huaibei, 235000 China; 3 College of Horticulture Science and Technology, Hebei Normal University of Science and Technology, Qinhuangdao, 066000 China; 4 Biotechnology Research Institute, Chinese Academy of Agricultural Sciences, Beijing, 100081 China

Seeds, the primary reproductive organs of most angiosperms, are essential to the plant life cycle because they store genetic material and supply nutrients for early seedling growth (He et al. 2026). However, seedlessness markedly improves fruit quality for fresh consumption and processing by increasing the edible proportion and eliminating hard seeds, making it one of the most valuable horticultural traits (Wang et al. 2021). Seedless fruits may occur naturally or be induced through hormone application, ploidy manipulation, or genetic engineering (Pang et al. 2025). Previous studies have demonstrated that parthenocarpy and stenospermocarpy are two distinct mechanisms underlying seedless fruit formation (Royo et al. 2018). In parthenocarpy, fruits develop from the ovary without pollination or fertilization, as observed in cucumber. In watermelon, seedlessness can be achieved either through conventional breeding of triploid plants or through the application of hormone analogs such as *N*-(2-chloro-4-pyridyl)-*N’*-phenylurea (CPPU). In contrast, stenospermocarpy involves successful pollination and fertilization followed by abortion of seed development due to defects in integument or endosperm formation. This process results in normal fruit enlargement with only rudimentary seed remnants, as seen in seedless grapes (Royo et al. 2018). With recent advances in genetic engineering, genome editing has become a feasible approach for generating seedless fruits, such as seedless watermelon (Jiang et al. 2024).

MADS-box genes encode a major family of transcription factors that regulate diverse physiological and developmental processes in plants (Favaro et al. 2025). Within the ABCDE model of floral organ development, the C-class gene *AG*, which governs stamen and carpel formation, and the D-class genes *STK* (previously *AGL11*), *SHP1*, and *SHP2*, which regulate ovule development, all belong to the AG subfamily of type II MADS-box genes in *Arabidopsis* (Pinyopich et al. 2003; Mizzotti et al. 2012). In addition, functional studies of the tomato D-class gene *SlMBP3/AFF* have shown that its mutation results in all-flesh fruit with non-liquefied locules and severely deformed seeds lacking a seed coat (Zhang et al. 2019; Huang et al. 2021). In grape, a single amino acid substitution (R197L) in VviAGL11 confers the stenospermocarpic seedless phenotype (Royo et al. 2018). Despite these findings, it remains unclear whether the functions of these genes are conserved in cucurbit crops.

Melon is an important horticultural crop in the Cucurbitaceae family and is closely related to watermelon, although their edible tissues differ anatomically. In watermelon, the flesh primarily originates from the fleshy placenta, whereas in melon, the edible portion consists mainly of the mesocarp and endocarp, which expand during fruit maturation as the placenta liquefies. Genetic resources for parthenocarpy in melon are limited and remain underutilized in commercial breeding. Moreover, seed coat formation often persists even after CPPU treatment. Therefore, developing seedless or low-seed melon varieties is an urgent breeding objective.

In this study, we performed a genome-wide identification of MADS-box genes in melon. A total of 46 MADS-box genes were identified, comprising 13 type I and 33 type II members (Table S1). The type II genes were further classified into 12 subfamilies (Fig. S1; Table S1). Phylogenetic analysis of the AG subfamily identified four melon genes belonging to this group (Fig. 1A). Among them, *MELO3C033521* showed the closest phylogenetic relationship with *AFF*, *STK*, and *VviAGL11*, with protein sequence similarities of 56.9%, 60.6%, and 88.3%, respectively (Fig. 1A). Based on this relationship and to facilitate functional analysis, this gene was designated *CmSLM* (*Cucumis melo solid-placenta and less-seed melon*). To reflect both its molecular homology and functional characteristics, it is referred to as *CmSLM/CmAGL11* throughout this study.

**Fig. 1:**
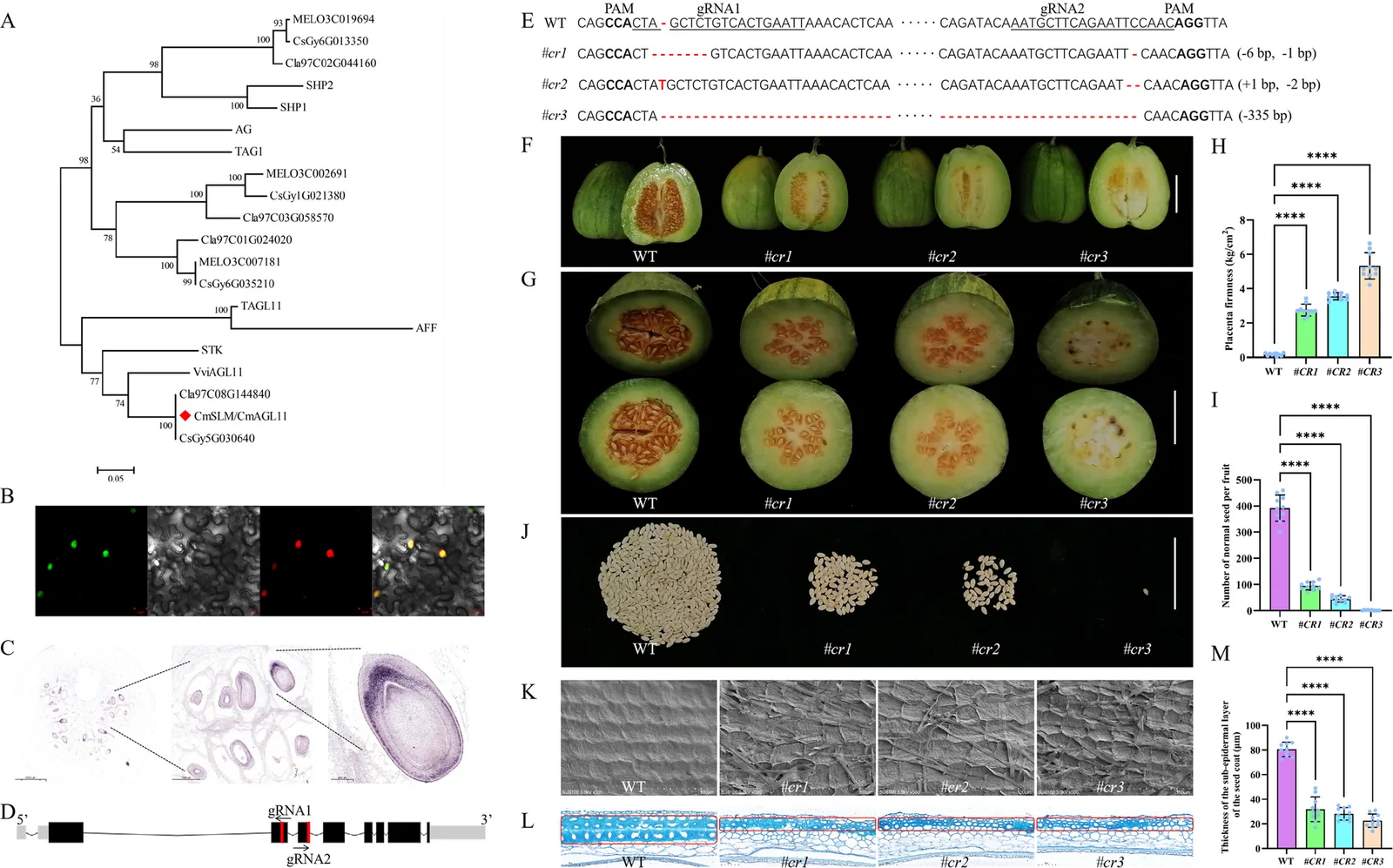
Creation of solid-placenta and less-seed melon via CRISPR-Cas9-mediated gene editing. **A** Phylogenetic analysis of AG subfamily MADS-box genes. **B** Subcellular localization of the CmSLM*/*CmAGL11-GFP fusion protein. Scale bar = 20 μm. **C** In situ hybridization analysis of *CmSLM/CmAGL11* mRNA in fruits at 5 days after pollination. **D** Gene structure and the two designed target sites of *CmSLM/CmAGL11*. **E** Editing outcomes of selected homozygous mutants. PAM sequences are shown in bold, and sgRNA target sequences are underlined. **F** Longitudinal section and **G** cross section of mature fruits. Scale bar = 5 cm. **H** Placental firmness, normal seed number per fruit (**I**), and seed production (**J**) in WT and three edited lines. Scale bar = 5 cm. **K** Scanning electron micrographs of mature dry seeds. Scale bar = 100 μm. **L** Paraffin section of seed coat cross-sections. The sub‑epidermal layer is outlined with a red rectangle. Scale bar = 50 μm. **M** Thickness of the seed coat sub‑epidermal layer. Values are means ± SD, n = 10. **** represents P < 0.0001 as determined by Student's *t*-test

Subcellular localization analysis showed that the CmSLM/CmAGL11-GFP fusion protein co-localized with the nuclear marker RFP-tagged HTB2 (Fig. 1B). Tissue-specific expression analysis revealed predominant expression in hermaphroditic flowers and fruits, with particularly high levels in the placenta (Fig. S2). Consistently, in situ hybridization of fruits demonstrated that *CmSLM/CmAGL11* transcripts were mainly localized in the placenta, with strong signals detected in developing seeds (Fig. 1C), suggesting a role in seed development. To further elucidate the function of *CmSLM/CmAGL11*, a CRISPR/Cas9 construct containing two sgRNAs targeting the third and fourth exons of *CmSLM/CmAGL11* was generated and introduced into the thin-skinned melon inbred line IVF105 via *Agrobacterium*-mediated transformation (Fig. 1D, S3). Five positive T_0_ transgenic lines were obtained, and mutations at both target sites were confirmed by Sanger sequencing. Three independent T_2_ homozygous mutant lines (*cr1*, *cr2,* and *cr3*) were selected for further analysis. The edited lines *cr1* and *cr2* carried frameshift mutations caused by base insertion or deletion at the first and second target sites, respectively, resulting in truncated proteins lacking the K-box domain (Fig. 1E, S4, S5). In contrast, *cr3* exhibited a large-fragment deletion that eliminated both conserved domains (Fig. 1E, S5, S6). Furthermore, *CmSLM/CmAGL11* transcript levels were significantly reduced in all three mutants (Fig. S6). Agronomic trait evaluation of the T_3_ generation showed no significant differences between mutant lines and WT in plant height or fruit development period (Fig. S7A, B). During fruit ripening, fruit weight and soluble solids content were comparable between mutant and WT fruits, whereas pericarp firmness was significantly higher in the mutant lines (Fig. S7C-E).

Internal examination of mature fruits revealed pronounced differences between the mutant lines and WT (Fig. 1F, G). The placental tissue of WT fruits was fully liquefied, whereas all mutants retained non-liquefied, solid placentas, resulting in a 15- to 30-fold increase in placental firmness (Fig. 1H). Mature WT fruits typically contained ~400 seeds, while seed numbers were significantly reduced across all three mutant lines (Fig. 1I, G, S8A). In particular, the *cr3* mutant produced only 0–3 normal seeds, accompanied by 13.5 ± 3.2 aborted seeds (Fig. 1I, G, S8A, S9). Both heterozygous edited plants and F_1_ progeny from crosses between the *cr3* mutant and WT displayed normal fruit phenotypes, indicating that the mutation is recessive. Compared to WT, mutant seeds exhibited thinner seed coats that were prone to cracking and displayed numerous longitudinal surface striations, together with a significant reduction in thousand-seed weight (Fig. S8B, S10). Scanning electron microscopy of mature dry seeds further showed that mutant seed coats possessed prominent cell wall protrusions and abundant band-like structures of variable length and width, in contrast to the smooth and regularly arranged epidermal cells observed in WT (Fig. 1K). Semi-thin sectioning revealed a substantial reduction in both size and thickness of the sub-epidermal layer in mutant seed coats, providing a structural basis for their increased brittleness (Fig. 1L, M). Collectively, these findings indicate that the melon *CmSLM/CmAGL11* gene is involved in regulating placental liquefaction and supporting normal seed development.

In conclusion, loss of *CmSLM/CmAGL11* function led to non-liquefied placentas and severely impaired seed development. In particular, large-fragment deletions produced predominantly solid placentas and near-seedless fruits with a phenotype resembling that of watermelon. Unlike CPPU-induced seedless melons, which retain empty seed coats, the solid-placenta, few-seeded melons generated in this study were produced through disruption of a MADS-box gene. Looking ahead, optimization of *CmSLM/CmAGL11* target sites, removal of exogenous vector sequences, and combination with CPPU treatment may enable the development of elite, truly seedless melon cultivars.

## Supplementary Information

Supplementary Material 1: Fig. S1. Phylogenetic analysis of MADS-box proteins from melon and *Arabidopsis thaliana*. The phylogenetic tree was constructed using the maximum likelihood method with 1,000 bootstrap replicates. Fig. S2. Spatiotemporal expression patterns (A) and relative transcript levels (B) of *CmSLM* in different melon tissues. Fig. S3. Schematic diagram of the CRISPR/Cas9 gene-editing vector used in this study. Fig. S4. Sanger sequencing chromatograms of the two *CmSLM* sgRNA target sites in the wild type and three edited lines. PAM sequences (red boxes) and target sequences (underlined) are indicated. Fig. S5. Protein length and conserved domains of the predicted *CmSLM* proteins in the wild type and three edited lines. Fig. S6. Molecular characterization of *CmSLM* in the wild type and three edited lines. A Genomic PCR analysis of Cm*SLM*. B RT-PCR analysis of Cm*SLM*; C qRT-PCR analysis of *CmSLM* in fruits at 5 days after pollination. Values are means ± SD of six biological replicates. **** indicates P < 0.0001 as determined by Student's *t*-test. Fig. S7. Evaluation of agronomic trait in the wild type and three edited lines. A Plant height. B Fruit development period. C Fruit weight. D Central soluble solids content. E Fruit pericarp firmness. Values are means ± SD (n = 10). * and **** indicate P < 0.05 and P < 0.0001, respectively, as determined by Student's *t*-test. Fig. S8. Statistical analysis of seed number per fruit (A) and 1000-grain weight (B) in the wild type and three edited lines. Values are means ± SD (n = 10). **** represent P < 0.0001 as determined by Student's *t*-test. Fig. S9. Aborted seeds in the wild type and three edited lines. Scale bars = 5 cm. Fig. S10. Mature seed morphology of the wild type and three edited lines. Scale bar = 1 mm.

Supplementary Material 2: Table S1. The information of the MADS-box gene in melon.

## Data Availability

All data are available in the manuscript. The datasets and materials of this study are available from the corresponding author upon reasonable request.
