# Supplementary material for: De novo creation of solid-placenta, few-seeded melon via targeted mutation of CmSLM/CmAGL11
Source: Mol Hortic. 2026 Nov 1;6:81. doi: 10.1186/s43897-026-00263-6 (PMC13723930; doi:10.1186/s43897-026-00263-6)
Supplement: Supplementary file 1 [file 43897_2026_263_MOESM1_ESM.docx]

**Supplementary Figures**

**Fig. S1**. **Phylogenetic analysis of MADS-box proteins from melon and *Arabidopsis thaliana*.** The phylogenetic tree was constructed using the maximum likelihood method with 1,000 bootstrap replicates.

**Fig. S2**. **Spatiotemporal expression patterns (A) and relative transcript levels (B) of *CmSLM* in different melon tissues.**

**Fig. S3**. **Schematic diagram of the CRISPR/Cas9 gene-editing vector used in this study.**

**Fig. S4**. **Sanger sequencing chromatograms of the two *CmSLM* sgRNA target sites in the wild type and three edited lines.** PAM sequences (red boxes) and target sequences (underlined) are indicated.

**Fig. S5**. **Protein length and conserved domains of the predicted *CmSLM* proteins in the wild type and three edited lines.**

**Fig. S6**. **Molecular characterization of *CmSLM* in the wild type and three edited lines.** **A** Genomic PCR analysis of Cm*SLM*. **B** RT-PCR analysis of Cm*SLM*; **C** qRT-PCR analysis of *CmSLM* in fruits at 5 days after pollination. Values are means ± SD of six biological replicates. **** indicates P < 0.0001 as determined by Student's *t*-test.

**Fig. S7**. **Evaluation of agronomic trait in the wild type and three edited lines. A** Plant height. **B** Fruit development period. **C** Fruit weight. **D** Central soluble solids content. **E** Fruit pericarp firmness. Values are means ± SD (n  =  10). * and **** indicate P < 0.05 and P < 0.0001, respectively, as determined by Student's *t*-test.

**Fig. S8**. **Statistical analysis of seed number per fruit (A) and 1000-grain weight (B) in the wild type and three edited lines.** Values are means ± SD (n  =  10). **** represent P < 0.0001 as determined by Student's *t*-test.

**Fig. S9**. **Aborted seeds in the wild type and three edited lines.** Scale bars = 5 cm.

**Fig. S10**. **Mature seed morphology of the wild type and three edited lines.** Scale bar = 1 mm.
